# Identifying Basketball Performance Indicators in Regular Season and Playoff Games

**DOI:** 10.2478/hukin-2013-0016

**Published:** 2013-03-28

**Authors:** Javier García, Sergio J. Ibáñez, Raúl Martinez De Santos, Nuno Leite, Jaime Sampaio

**Affiliations:** 1 University of Extremadura, Faculty of Sports Sciences, Cáceres, Spain.; 2 University of the Basque Country (UPV/EHU), Department of Physical Education and Sport, Vitoria–Gasteiz, Spain.; 3 Research Center in Sports Sciences, Health Sciences, and Human Development (CIDESD).

**Keywords:** Performance analysis, game-indicators, win/lose, basketball

## Abstract

The aim of the present study was to identify basketball game performance indicators which best discriminate winners and losers in regular season and playoffs. The sample used was composed by 323 games of ACB Spanish Basketball League from the regular season (n=306) and from the playoffs (n=17). A previous cluster analysis allowed splitting the sample in balanced (equal or below 12 points), unbalanced (between 13 and 28 points) and very unbalanced games (above 28 points). A discriminant analysis was used to identify the performance indicators either in regular season and playoff games. In regular season games, the winning teams dominated in assists, defensive rebounds, successful 2 and 3-point field-goals. However, in playoff games the winning teams’ superiority was only in defensive rebounding. In practical applications, these results may help the coaches to accurately design training programs to reflect the importance of having different offensive set plays and also have specific conditioning programs to prepare for defensive rebounding.

## Introduction

Traditionally, preparing basketball teams to succeed in competition was a complex process that was based on players’ fitness levels and anthropometric characteristics (Sampaio et al., 2010). Nowadays, coaches prepare the competition and training process using notational analysis with the scope of improving both the team’s and the players’ performances (Hughes and Franks, 2004; Ortega et al., 2009; Leite et al., 2009). Notational analysis has been described as the process of recording, treatment and diagnostics of events that take place in competition (Drust, 2010). Performance analysis in basketball is currently an essential tool for coaches and technical staff. This analysis method allows them to collect reliable information about their opponents, competition and, mainly, their own team.

Basketball has been one of the most analyzed sports through notational analysis (Lorenzo et al., 2010). Game-related statistics are very popular among coaches, players and researchers and have been used to improve understanding of game performance in different contexts (Gómez et al., 2010; Gómez et al., 2009; Ibáñez et al., 2008; Sampaio and Janeira, 2003).

The investigation in this area has been focused, traditionally, on men’s basketball teams (Ibáñez et al., 2003; Trninić et al., 2002). These topics have been widely studied and some important trends have been found: fields goals and defensive rebounds are the game performance indicators that best discriminate between winning and losing teams in basketball (Ibáñez et al., 2003; Ittenbach and Esters, 1995; Karipidis et al., 2001). Sampaio et al. (2010) suggest that winning teams performance is due to the achievement of more opportunities to attempt field-goals and also to the improvement in the decision making of the players of the winning teams as well as a better strategic and tactical environment. More recent studies have been exploring contextual factors such as game location (home and away), game type (regular season and playoff), game final score differences (close, balanced and unbalanced games), players’ gender (men and women), level of competition (Euroleague, National Basketball Association, etc.) and age (senior and junior) to establish better understanding of performance analysis (Lorenzo et al., 2010).

The study of game performance as a function of the differences in the final score of the game is becoming an important variable to consider. Available literature has analyzed categories separately (Sampaio and Janeira, 2003; Gómez et al., 2008). In balanced games (equal or below 12 points) the game performance indicators that discriminated between winners and losers were defensive rebounds (Gómez et al., 2008), missed 3-point field goals (Gómez et al., 2009), field goals percentages and defensive rebounds (Janeira et al., 1996) and in unbalanced games (between 13 and 28 points of final score difference). Sampaio and Janeira (2003) concluded that losing teams performed poorly in all performance indicators. Gómez et al. (2008) found that successful 2 points field-goals, defensive rebounds and assists discriminated between winning and losing teams.

Conversely, only a few studies have analyzed performance in function of a competition phase and never with high-level basketball competition. In fact, most of professional leagues are organized in two phases: firstly, all the teams compete against each other twice in a double round, with the aim of add up victories to classification. Finally, only the top classified teams (usually the best 8 teams of the regular season) play-off for the championship. The qualifying round is organized in such a way that best classified teams compete against the worst classified team and so on in a knockout round the best of three or five games. Thus, winning a game has a different importance: while in the regular season you can lose three games in a row and remain in competition, if you lose two of three, or three of five playoff games in a row you will be eliminated. Sampaio and Janeira (2002) studied Portuguese Professional Basketball League, which is a lower level competition according to the International Basketball Federation (Gómez et al., 2008). They pointed out that in the playoff games there were less ball possessions (71.16) than in the regular season games (74.75). Hence, the game rhythm was slower with a direct impact on the points per game, reducing the final score. There are no more studies inspecting this contrast between regular season and playoff games. Therefore, the aim of the present study was to identify basketball game performance indicators, which best discriminate between winners and losers in regular season and playoff games.

## Material and Methods

### Sample and variables

Archival data were obtained from the Asociación de Clubs de Baloncesto (ACB), the Spanish professional league, for the 2007–2008 season. The collected data were gathered in live by ACB professional technicians. 323 games were analyzed, 306 from regular season and 17 from playoffs. Data were selected from the official boxscores of ACB. The game-related statistics included free-throws, 2 and 3 point field goals (both successful and unsuccessful), offensive rebounds, defensive rebounds, assists, steals, turnovers, received and committed fouls and blocks received and committed. The coefficient of agreement of these variables was high (kappa above .92) (Ibáñez et al., 2009; Sampaio et al., 2010). The reliability data test was not carried out because the ACB technicians have their own reliability procedures carried in real time with the referees’ boxscores. Afterwards, the values were normalized to 100 ball possessions (Sampaio and Janeira, 2003) allowing to eliminate the game rhythm effect. This procedure allows generalizing the results and comparing with other studies. For example, the performance of a team that makes 40 fields-goals in a 70 possession game must be different from the performance of another team that makes 30 fields-goals in the same 70 possession game. Ball possessions were calculated by the following equation (BP = Attempted field goals – offensive rebounds + turnovers - 0.4 × Attempted free throws) (Oliver, 2004).

### Statistical Analysis

Prior to the inferential statistical analysis the sample was categorized into three groups using the cluster of *k*-means method (Norušis, 2005; Sampaio et al., 2004). This algorithm aims to establish a classification of the objects into a *K* number of groups based on similar characteristics (Bishop, 1995). The clustering is done by minimizing the sum of squares of distances between data and the corresponding centroid group which represents the arithmetic mean for each dimension separately over all the points in the cluster. All games were classified into three groups: 65.9% of the sample fitted in group 1 with final score differences equal or below 12 points (balanced games), 31.8% of the sample fitted into group 2 with final score differences between 13 and 28 points (unbalanced games), and 2.3% of the sample were classified in another group of games with final score differences above 28 points (very unbalanced games). Due to their poor relevance, this last group was omitted from subsequent analyses. An independent measures ANOVA was used to identify differences between winners and losers for each regular season and playoff games. Also, a discriminant analysis was performed to identify which of the game-related statistics best discriminated winning and losing teams (Ntoumanis, 2001) for each group of games. The structural coefficients (*SC*) were considered relevant when above |0.30| and were used to identify the variables that best predicted its belonging to the group of winners (Pedhazur, 1982). Statistical significance was set at p≤0.05. The statistical analyses were performed using SPSS software release 15.0.

## Results

The means and standard deviations of game-related statistics for ACB regular season and playoffs are presented in Table 1. A discriminant function was performed to identify differences between winning and losing teams in regular season games. This function was statistically significant (*p*≤0.001) with a canonical correlation of 0.71 (Λ= 0.48) and reclassification of 86.7%. The structure coefficients from the function reflected an emphasis on assists, defensive rebounds, successful 2 and 3 point field-goals (Table 1). In playoff games, a statistically significant discriminant function was also found (*p*≤0.001), with a canonical correlation of 0.92 (Λ= 0.15) and reclassification of 86.8%. However, there were no variables emphasized by the structure coefficients (Table 1).

Table 2 presents the analysis performed for balanced games (final score differences under 12 points). The discriminant function obtained was statistically significant (*p*≤0.001) and had an overall percentage of successful reclassification of 80.5%. For these games, the canonical correlation was 0.63(Λ= 0.59). The structure coefficients from the function reflected an emphasis on assists, defensive rebounds and successful 2 point field-goals (Table 2). In playoff games, the discriminate analysis was not statistically significant although the univariate ANOVA revealed differences between winning and losing teams in defensive rebounds and successful 2 point field-goals (Table 2).

The results from unbalanced games (13 to 28 points) are presented in Table 3. The discriminant function obtained was statistically significant (*p*≤0.001) and had an overall percentage of successful reclassification of 80.5%. For these games, the canonical correlation was of .91 (Λ= 0.16). The structure coefficients from the function reflected an emphasis on assists (Table 3). In the playoffs, the univariate ANOVA allowed identifying differences between winning and losing teams in successful 3 point field-goals and defensive rebounds (Table 3).

## Discussion

The aim of the present study was to identify game related statistics, which best discriminate between winners and losers in regular season and playoff games. Different season phases have different types of games, playoff games have different characteristics (only games confronting the best teams, several consecutive games between teams, knockout phase) which require different strategies and tactics. Available research in this topic is very scarce and has not been used with high level teams. Nonetheless, it seems clear that playoff games, due to their importance for the final classification, the need of victories or the level of opponents, have a lower number of ball possessions per game, and a lower game pace (Sampaio and Janeira, 2002). Having this assumption, the results of this study confirm that winning and losing teams used different strategies and tactics in regular season and playoff games.

When analyzing all regular season games, it was possible to identify a small subset of game performance indicators that discriminated winning and losing teams (assists, defensive rebounds, successful 2 and 3 point field-goals). The development of offensive set plays by all the teammates allowed a better process of perception, decision and execution, and best performance by players from the winning teams (Gómez et al., 2008). Assists are an indicator of teamwork, and give more opportunities to score and win (Hoofler and Payne, 1997).

Assists and turnovers are not only related with the technical capacity of the players but the decision and perception process, which are related with, for example, team maturity or timing, which are very important to score a basket (reading defenses and knowing which is the better moment to pass the ball to a teammate). On the other side, several studies showed that elite players have a higher level of physical fitness (Royal et al., 2006), which contributes to better decision making in the game. Besides, Lyons et al. (2006), concluded that the higher physical fitness level may allow these players to maintain the accuracy and performance level in the passing skill compared to less experienced players. For all these reasons, it was expected that successful 2-point field goals discriminated between winners and losers. Moreover, Trninić et al. (2002) suggested that worst teams are less accurate due to poor tactics.

Winning teams’ performances could also be discriminated by their higher number of defensive rebounds (Gómez et al., 2008; Ibáñez et al., 2003; Trninić et al., 2002). This game statistic is important to enhance the chances of the winning teams at scoring and winning while limiting the opposition’s chances to score (Hoofler and Payne, 1997). The players have an individual responsibility to block the opponent and get the rebound and, supporting this idea, Trninić et al. (2002) suggest that less experienced players performed worst in this element, allowing the opponents to capture more offensive rebounds. On the other hand, defensive rebounds are associated with a better defensive level. A good defense forces the attacking team to take bad shoots, of low effectiveness, which are converted into rebounds. Besides, defensive rebounding avoids the opposite team to get a new possession, closer to the basket (Trninić et al., 2002).

According to Ibáñez et al. (2009), defensive rebounds have an indirect influence on i) game rhythm, more rebounds would mean more opportunities for fast breaks, which are initiated after defensive rebounds, principally, after steals or a basket (Refoyo et al., 2010); ii) players‘ morphologic characteristics, bigger players have increased rebounding chances (Papadimitriou et al., 1999); iii) technical and tactical preparation, centers have to pivot, block and secure space for rebounding; and iv) physical conditioning, especially strength. Therefore, coaches should establish game strategies to achieve defensive rebounds or put on the court players with a high rebounding capacity.

Finally, successful 3-points field goals allowed to identify the differences between teams. Several studies were focused on the importance of these game performance indicators previously (Durkovic et al., 2005; Karapidis et al., 2001; Sampaio and Janeira, 2003). In recent years, it seems clear that long distance shooting has been acquiring greater influence on the game dynamics creating more spaces for players closer to the basket due to the higher accuracy of three point shooters. Those players are becoming specialists, focusing their performance on long distance shooting. In successive matches, e.g. three in a row, 3-point field-goals discriminate between winning and losing teams (Ibáñez et al., 2009). The authors concluded that when fatigue appears, best players perform better than worst ones. In fact, in juniors and women competition, long distance shooting predicts winning or losing the games (Sampaio et al., 2004).

In playoff games, there were no variables emphasized by the structure coefficients. The available literature in basketball playoff games is scarce and does not focus on A.C.B. League. However, it is known that playoff games have less ball possessions compared with regular season ones and that the game pace is slower (Sampaio and Janeira, 2002). In the 2008 Olympics, the USA team had 11 more ball possessions than the rest of the teams (81.1 vs 70.7) (Pelton, 2008). This increase in the game pace resulted in more ball recoveries of the USA team (Sampaio et al., 2010). Playing at a higher pace leads to more unforced errors and coaches try to minimize errors (turnovers) in most important games, such as finals or playoffs. Thus, it seems clear that in playoff games there are fewer actions due to a lower game rhythm and every action becomes more important for the outcome of the game.

In regular season balanced games, the analysis emphasized assists, defensive rebounds and successful 2 point field-goals (Gómez et al., 2008). Only successful 3 point field goals were deemphasized when compared to all games. It seems obvious that differences between teams in successful 3 point field goals will have major implications for the final score differences. However, results pointed out that in balanced games the teams had to be more effective in short distance field goals to win the match. In playoff games the differences between winning and losing teams were in defensive rebounds and successful 2 point field-goals. Results showed that assists were less important in playoff games. Therefore, it might be possible that in these decisive games teamwork is not so frequent and that the best players systematically decide ball possessions.

In regular season unbalanced games, assists were important to differentiate winning and losing teams (30.13 vs 26.52). Winning teams demonstrated better adaptation to use ball possessions and tried to achieve easy baskets, probably using planned strategies of 2 on 2 or 3 on 3 (Gómez et al., 2008). Likewise, winning teams reduced the assists of their opponents (20.88 in unbalanced games vs 22.26 in balanced games) and showed a better defensive performance. In fact, a good defense avoids the opponents’ assists and forces poor shots. In playoffs, the differences between winning and losing teams were in defensive rebounds and the games ended unbalanced by the differences in successful 3 point field-goals. A team would win an unbalanced game if able to perform effectively in 3 points field goals (16.29 against 8.45 in unbalanced games compared with 15.92 against 15.90 in balanced games).

In conclusion, our results indicate that winning and losing teams played different in regular season and playoff games. Overall, the regular season games were dominated by the importance of assists showing the importance of teamwork during this phase. On the contrary, the playoff games were dominated by the importance of effective defensive rebounding. As a consequence of a slower game pace and a higher competitive importance of the game itself this might reduce successful field-goals and free throws. Defensive rebounds′ importance increases to secure these unsuccessful shots. In practical applications, these results may help coaches to design training programs more accurately taking into account the importance of having different offensive set plays (involving more players in regular season and fewer players in playoffs) and specific conditioning programs to prepare for defensive rebounding.

As limitation of the study, it seems clear that quantitative analysis is not enough to understand what happens in matches. Qualitative complementary analysis is necessary to explain why and how.

## Figures and Tables

**Table 1:** Means, Standard deviations, and Discriminant Analysis Structure Coefficients (SC) from game performance indicators by winning and losing teams on ACB 2007/078 Regular Season and Play-Offs.

	Regular Season(N=306)					Play-offs (N=17)				

	Winning teams		Losing teams			Winning teams		Losing teams		

	*M*	*SD*	*M*	*SD*	*CCE*	*M*	*SD*	*M*	*SD*	*CCE*

2 point successful	38.63	7.59	33.09	7.32	.36^†^	41.23	6.52	35.98	6.45	.17
2 point unsuccessful	31.41	8.89	34.75	8.92	−.18	30.84	9.21	37.09	7.11	−.16
3 point successful	16.71	5.46	13.46	4.90	.30^†^	16.05	5.45	13.27	5.29	.11
3 point unsuccessful	25.91	7.20	26.56	7.65	−.04	28.47	6.23	30.62	6.33	−.07
Free-throw successful	33.75	15.09	28.88	13.03	.16	44.03	19.18	34.70	12.19	.12
Free-throw unsuccessful	9.98	6.08	9.33	5.82	.05	11.40	4.42	12.28	5.12	−.04
Defensive rebounds	44.50	8.92	37.71	8.33	.38^†^	48.63	10.14	38.52	6.54	.26
Offensive rebounds	19.57	7.42	19.34	7.11	.01	19.86	7.24	22.61	8.67	−.07
Assists	27.90	7.66	21.40	6.13	.45^†^	29.32	7.27	26.48	6.36	.09
Steals	17.06	6.11	14.08	5.36	.25	16.43	4.96	15.31	5.77	.04
Turnovers	24.41	6.42	26.77	6.54	−.17	25.44	8.00	24.44	6.21	.03
Blocks commited	5.89	3.67	4.66	3.21	.17	7.95	5.06	4.84	3.99	.11
Blocks received	4.81	3.39	5.63	3.42	−.11	5.47	4.97	7.47	4.78	.15
Dunks	4.22	3.64	2.59	2.70	.24	6.50	3.72	4.52	3.44	−.09
Fouls commited	41.79	8.99	41.27	8.79	.02	47.89	9.83	47.40	9.78	.12
Fouls received	43.45	11.81	40.59	10.42	.12	50.69	14.35	45.61	10.20	.01
Wilks Lambda					.48^*^					.15^*^
Eigenvalue					1.05					5.52
Canonical Correlation					.71					.92

† SC ≥|0.30|;

* p≤.001

**Table 2:** Means, Standard Deviation, Discriminant analysis structure coefficients (SC) and Anova From game performance indicators by win and lose in balanced games in function of phase season ACB 2007/08 season.

	Regular Season (n= 203)					PlayOff (n= 11)				

	Winning teams		Losing teams			Winning teams		Losing teams		

	*M*	*SD*	*M*	*SD*	*SC*	*M*	*SD*	*M*	*SD*	*Sig.*

2 point successful	37.95	7.39	33.63	7.50	.35^†^	42.19	7.20	35.82	6.24	^‡^
2 point unsuccessful	32.44	8.80	34.14	8.58	−.11	31.16	7.83	36.06	6.70	
3 point successful	15.94	5.46	14.43	4.88	.17	15.92	5.98	15.90	4.34	
3 point unsuccessful	26.54	7.26	26.68	7.26	−.01	28.56	7.32	29.11	5.20	
Free-throw successful	35.34	16.05	30.00	14.00	.21	43.52	22.13	36.11	13.07	
Free-throw unsuccessful	10.46	6.24	9.33	5.55	.11	11.66	5.03	12.14	4.66	
Defensive rebounds	44.47	9.08	39.41	8.04	.35^†^	47.77	10.27	38.93	6.95	^‡^
Offensive rebounds	19.24	7.21	19.14	6.91	.00	20.29	7.91	20.62	6.06	
Assists	26.52	7.12	22.26	6.27	.38^†^	28.79	7.45	26.98	7.04	
Steals	16.46	5.77	14.16	5.34	.25	14.94	4.92	15.01	3.29	
Turnovers	24.69	6.34	26.00	6.39	−.12	24.53	5.70	23.04	5.75	
Blocks commited	5.97	3.66	4.77	3.07	.21	6.81	4.22	4.60	3.53	
Blocks received	4.96	3.29	5.66	3.45	.19	5.09	4.32	6.57	4.29	
Dunks	4.06	3.64	2.83	2.78	.23	5.75	3.70	4.86	3.65	
Fouls commited	42.75	9.32	42.48	8.91	.01	47.37	10.74	47.80	9.26	
Fouls received	45.04	12.30	41.24	10.79	.19	50.65	15.25	45.85	11.61	
Wilks Lambda			.59^*^							
Eigenvalue			.68							
Canonical Correlation			.63							

† SC ≥|0.30|;

* discriminant analysis (p≤.05);

‡ univariate differences (p≤.05)

**Table 3:** Means. Standard Deviations, Discriminant analysis structure coefficients (SC) and ANOVA From game performance indicators by win and lose in Unbalanced games in function of phase season ACB 2007/08 season.

	Regular Season (n=97)					Play Off(n=6)				

	Winning teams		Losing teams			Winning teams		Losing teams		

	*M*	*SD*	*M*	*SD*	*SC*	*M*	*SD*	*M*	*SD*	*Sig.*

2 point successful	39.84	7.37	32.50	7.02	.23	39.47	5.18	36.28	7.40	
2 point unsuccessful	29.59	8.89	36.19	9.25	−.16	30.25	12.16	39.00	8.12	
3 point successful	17.89	5.10	11.66	4.43	.29	16.29	4.84	8.45	2.95	^‡^
3 point unsuccessful	24.97	6.86	27.09	8.22	−.06	28.33	4.09	33.40	7.73	
Free-throw successful	31.69	14.05	27.82	11.05	.07	44.97	14.00	32.10	11.00	
Free-throw unsuccessful	9.13	5.54	9.63	6.35	−.01	10.91	3.38	12.54	6.36	
Defensive rebounds	44.77	8.80	35.07	7.51	.26	50.20	10.66	37.75	6.25	^‡^
Offensive rebounds	19.98	7.76	20.32	7.84	−.01	19.09	6.46	26.28	11.91	
Assists	30.13	7.18	20.88	5.87	.32^†^	30.29	7.52	25.58	5.37	
Steals	17.94	6.47	14.20	5.37	.14	19.16	4.19	15.86	9.19	
Turnovers	24.03	6.66	27.87	6.82	−.12	27.10	11.62	27.01	6.71	
Blocks commited	5.94	3.55	4.60	3.64	.15	10.04	6.20	5.27	5.08	
Blocks received	4.73	3.89	5.83	3.49	−.06	6.17	6.39	9.13	5.59	
Dunks	4.69	3.58	2.50	2.76	.15	7.89	3.64	3.90	3.25	
Fouls commited	40.79	8.74	39.75	8.99	.02	48.84	8.76	46.68	11.57	
Fouls received	41.13	11.31	40.28	10.03	.01	50.77	13.91	45.17	7.95	
Wilks Lambda			.16^*^							
Eigenvalue			4.90							
Canonical Correlation			.91							

† SC ≥|0.30|;

* discriminant analysis (p≤.05);

‡ univariate differences (p≤.05)
